# The Use of Retrieval Practice in the Health Professions: A State-of-the-Art Review

**DOI:** 10.3390/bs15070974

**Published:** 2025-07-17

**Authors:** Michael J. Serra, Althea N. Kaminske, Cynthia Nebel, Kristen M. Coppola

**Affiliations:** 1 Department of Medical Education, School of Medicine, Texas Tech University Health Sciences Center, Lubbock, TX 79430, USA; 2 Department of Surgery, Indiana University School of Medicine, Indianapolis, IN 46202, USA; abauerns@iu.edu; 3 Department of Psychiatry and Behavioral Neuroscience, St. Louis University School of Medicine, St. Louis, MO 63104, USA; cynthia.nebel@health.slu.edu; 4 Department of Psychiatry, Rutgers Robert Wood Johnson Medical School, Piscataway, NJ 08854, USA

**Keywords:** retrieval practice, health professions education, applied cognitive psychology, self-regulated learning, test-enhanced learning

## Abstract

Retrieval practice, or the active recall of information from memory, is a highly effective learning strategy that strengthens memory and comprehension. This effect is robust and strongly backed by research in cognitive psychology. The health professions—including medicine, nursing, and dentistry—have widely embraced retrieval practice as a learning and study tool, particularly for course exams and high-stakes licensing exams. This state-of-the-art review examines the historical development, current applications, and future directions for the use of retrieval practice in health professions education. While retrieval-based learning has long been used informally in these fields, its formal recognition as a scientifically supported study method gained momentum in the early 2000s and then saw a surge in both research interest and curricular adoption between 2010 and 2025. This historical review explores the key factors driving this growth, such as its alignment with assessment-driven education and the increasing availability of third-party study resources that rely on retrieval practice as a guiding principle. Despite its proven benefits for learning, however, barriers persist to its adoption by students, including in the health professions. This article discusses strategies for overcoming these challenges and for enhancing retrieval practice integration into health professions curricula.

## 1. Introduction

Retrieval practice (sometimes called ‘the testing effect’, ‘retrieval-based learning’ or ‘test-enhanced learning’) describes the finding that retrieval, or the process of recalling information from the mind, enhances one’s learning and memory. Extensive research in cognitive psychology highlights the effectiveness of retrieval practice for enhancing retention and comprehension (Agarwal et al., 2008; Karpicke, 2017). Practice tests and other assessments not only reinforce learning but also provide valuable feedback to guide restudy (Ariel & Karpicke, 2018).

The health professions—including medicine, nursing, and dentistry—have widely adopted retrieval practice as an effective method for learning and studying, particularly in preparation for high-stakes and licensing exams. This review explores how faculty, students, learning specialists, licensing bodies and educational resource developers have integrated the use of retrieval practice into health education. We have taken a “state-of-the-art” review approach, presenting a historical record which includes the inconsistent endorsement of retrieval practice in these fields prior to 2010, the significant adoption and proliferation of retrieval practice as a topic of research and as a major method of learning from 2010 to the present (2025) and some potential future directions for research and the use of retrieval practice in the health professions going forward.

In the present review, we use the term “learning” to mean any measurable change in behavior following an event indicating a change in knowledge, skills, or attitude (cf. American Psychological Association, 2018). We recognize the breadth of this definition, but the term is defined in many different ways across fields (Barron et al., 2015), so a definition that covers most versions of the concept will necessarily be vague.

### 1.1. Retrieval Practice

The first experiments investigating the effects of retrieval practice on memory were conducted by Edwina E. Abbott in 1909 (Abbott, 1909). In her master’s thesis, she concluded, “recall is always an aid in the learning process” (p. 25). The topic was reported on intermittently during the 20th century (e.g., Gates, 1917; Glover, 1989; McDaniel & Fisher, 1991; Spitzer, 1939; see also Roediger & Karpicke, 2006b) but saw a resurgence in 2006 when Roediger and Karpicke published, “Test-enhanced learning: Taking memory tests improves long-term retention”. They concluded that “[t]esting is a powerful means of improving learning, not just assessing it.” (p. 249). In the two decades since, interest in the study and application of retrieval practice has only grown (Pan et al., 2024).

The variety of terms used to refer to this effect reflects the nuance in understanding of the phenomena spearheaded by cognitive psychologists in the past two decades. Early reports on the effect in the 2000s used “the testing effect” or “test-enhanced learning”, wherein the emphasis was on the finding that people who were tested on material had improved long-term memory and understanding compared to people who simply re-read the same material (e.g., Agarwal et al., 2008; McDaniel et al., 2007; Roediger & Karpicke, 2006a). Research using the terms “retrieval practice” or “retrieval-based learning”, on the other hand, emphasized the process of retrieval and the factors that affect it (prior knowledge, feedback, format, etc.; e.g., Ariel & Karpicke, 2018; Karpicke, 2012). For example, if a learner completes a practice test, but performs poorly on it, would they still benefit from retrieval? Research indicates that the learner will benefit from retrieval practice, independent of their overall performance, so long as their performance is off the floor (Chan et al., 2024). Thus, research in cognitive psychology tends to refer to “retrieval practice” and focuses on the underlying cognitive processes, while “the testing effect” is still commonly used outside of the field to describe the educational context.

The nuanced distinctions within the retrieval practice literature can have important implications for the application of these findings to health professions education. For example, medical students make heavy use of flashcard software and multiple-choice question banks to prepare for course and licensing exams, without much regard for the specific cognitive processes engaged by these different forms of retrieval practice. Whereas flashcards promote repeated exposure to discrete facts, medical licensing exams involve complicated, multiple-choice questions that present case-based vignettes and require deep, applied reasoning to choose the best option among many plausible lures (Kolomitro et al., 2020; Little et al., 2019). Retrieval practice will be most beneficial when the type of retrieval practice matches the processing required on the exam (i.e., “transfer-appropriate processing”, see Morris et al., 1977), so engaging in retrieval via practice questions might produce more relevant retrieval practice.

### 1.2. The Growth of Retrieval Practice in Research and Applied Settings

According to Pan et al. (2024), 1215 peer-reviewed articles addressing “the testing effect”, “retrieval practice”, or “test-enhanced learning” were published from 1999 to 2022 across disciplines and domains. The popularity of retrieval practice within cognitive psychology, and its robust application to educational settings outside of that field, is likely due to three factors. First, it is a reliable and robust effect. In the work by Roediger and Karpicke (2006a), the effect sizes ranged from *d* = 0.31 to *d* = 1.26. Subsequent meta-analyses of the testing effect and retrieval practice found a reliable medium effect size of testing (*g* = 0.50; Rowland, 2014), medium effect sizes when looking at the transfer of test-enhanced learning (*d* = 0.40; Pan & Rickard, 2018) and a medium effect size in applied classroom settings (*g* = 0.50; Yang et al., 2021). It can be found across a variety of materials, retention intervals and test-types, and it requires a small sample size (20–40 per cell) to detect (Agarwal et al., 2021). Despite some initial evidence to the contrary (Van Gog & Sweller, 2015), retrieval practice benefits learning for complex materials (Karpicke & Aue, 2015; Rawson, 2015), including complicated health professions content such as physiology (J. Dobson et al., 2018), chronic wasting disease (Kriechbaum & Bäuml, 2024) and the aerobic production of adenosine triphosphate (J. L. Dobson et al., 2019). Individual differences do not seem to moderate the effect (cf. de Lima & Buratto, 2024), nor does students’ prior level of knowledge for a topic (cf. Buchin & Mulligan, 2023; Cogliano et al., 2019). Put simply, it is a powerful effect that is easy to study. Second, when introduced to a broader audience as “the testing effect”, this label provided a simple, yet counterintuitive, explanation for the effect. “Taking a memory test not only assesses what one knows, but also enhances later retention, a phenomenon known as the testing effect.” (Roediger & Karpicke, 2006a, p. 249). This framing describes memory phenomena in relatable and accessible terms (i.e., “testing” is a familiar concept to many, but “retrieval” or “recall” may be perceived as cognitive jargon), and turns the assumption that tests are used purely for assessment, rather than learning, on its head. This variation from the norm facilitates a third reason why this effect has received a significant amount of attention outside of just cognitive psychology: many educational fields rely heavily on assessment. For example, as early as 2008, Larsen, Butler and Roediger highlighted the benefits of testing and the ease with which medical education, a field that “has focused extensively on assessment issues”, could apply the testing effect to the education of its students (Larsen et al., 2008).

While cognitive psychologists continue to debate and dissect the cognitive processes and phenomena involved in retrieval, that type of nuance is unlikely to be found in the fields in which this research is applied. Thus, while we are reporting on the use of retrieval practice and retrieval-based learning in the health professions, we include papers that refer to the testing effect or test-enhanced learning, as well as earlier papers and other publications which used none of those terms but did involve forms of retrieval practice such as practice questions (especially in the health professions).

### 1.3. Why Have the Health Professions Embraced Retrieval Practice?

Several aspects of education in the health professions have made them a “best-case scenario” for applying retrieval practice and seeing clear benefits from its use. First, the health professions all require students to learn a high volume of very difficult content in a relatively short amount of time, while juggling other personal and professional demands (e.g., trying to gain research experience in a laboratory outside of curricular responsibilities), all while under intense pressure to obtain high scores in courses and to earn high or passing scores on national licensing exams (e.g., Rogers et al., 2016). Second, students of the health professions are typically very highly motivated to perform well in their courses and on licensing exams and are often seeking ways to improve their performance or make their studying more efficient (e.g., Lawrence et al., 2023; Matsko & Cervantes, 2024). Third, despite the very high admission standards for study in the health professions, a large portion of students begin their training in a health profession lacking strong study skills and habits (e.g., Ahmed et al., 2021; Didarloo & Khalkhali, 2014; Ezeala & Siyanga, 2015; Sisa et al., 2023). Self-regulated learning is a common name for a suite of skills which one uses to be “metacognitively, motivationally, and behaviorally proactive in the learning process” (Zimmerman & Pons, 1986). A common measure of students’ skills in self-regulated learning is the Learning and Study Strategies Inventory (LASSI), which includes ten subscales: anxiety, attitude, concentration, information processing, motivation, selecting main ideas, self-testing, test strategies, time management, and using academic resources (Weinstein et al., 1987; Weinstein et al., 2002). One study found half of medical students to be below the 50th percentile on several of the LASSI subscales (O’Sullivan et al., 2024). As well, some studies have found significant correlations between medical students’ scores on the LASSI subscales of anxiety, information processing, motivation, selecting main ideas, and test strategies with their performance on biomedical sciences content, anatomical sciences content, and on national licensing exams (Khalil et al., 2017, 2018, 2020). Fortunately, study skills interventions can improve students’ study skills as measured by the LASSI (Sisa et al., 2023). Fourth, although retrieval practice is just one tool that students of the health professions can utilize to maximize their learning, it is fairly simple to implement (by students, faculty and software) while being highly effective for enhancing memory and comprehension while reducing forgetting over time (e.g., Agarwal et al., 2021; Ariel & Karpicke, 2018; Donker et al., 2022). It also provides objective feedback on what information the students know or do not know (e.g., Ariel & Karpicke, 2018; England & Serra, 2012; Serra & Ariel, 2014), making retrieval practice a valuable component of self-regulated learning.

Most health profession curriculums include a mix of classroom-based instruction, seminars, laboratory/practical courses and—especially in the later phases of training—hands-on experience treating actual patients (Alishahedani et al., 2019; Barrison et al., 2024). These programs also formally and informally involve a heavy use of “third-party” resources: the written summaries, instructional videos, practice question banks, study software and other learning materials created by publishing companies, test-preparation companies and health education experts to supplement the curriculum. These third-party resources are sometimes referred to as the “parallel curriculum” in the health professions (Barrison et al., 2024). Students of the health professions often become overwhelmed by “resource fatigue” (Alishahedani et al., 2019). Besides the large volume of educational resources available to them, students in these fields must also navigate complicated (and potentially conflicting) advice from faculty, learning specialists, peers, tutors and online “experts” about these resources and ways to study effectively.

Although effectively using retrieval practice is just one component of self-regulated learning, it exemplifies the kind of evidence-based strategy that supports the development of lifelong learning skills—skills essential for success in the health professions. Given the arduous complexity, pace and volume of health education, students must be proactive, reflective and strategic in how they learn. Retrieval practice aligns with these demands: it not only strengthens memory and understanding but also fosters greater metacognitive awareness, helping students identify gaps in their knowledge and adjust their studying accordingly. As such, its integration into health professions education supports not just academic success, but also the formation of adaptive, self-directed professionals prepared for the demands of continuous learning throughout their careers (Artino et al., 2015).

### 1.4. The Present Review

A state-of-the-art review is a chronological overview of the evolution of knowledge about a phenomenon, highlighting current understanding, the historical developments that shaped it and future research directions. It synthesizes key turning points in the field to offer a modern, forward-looking perspective on the topic, informed by the past and present understanding of the topic (Barry et al., 2022; Grant & Booth, 2009). Accordingly, our review discusses the past, present and future use of retrieval practice as a learning and study method in the health professions.

The health professions have strongly embraced and applied findings from cognitive psychology (including retrieval practice and several other empirically supported methods for improving learning) for the training of their students. These fields are therefore not only an informative case study for how the science of learning can be successfully applied to actual curriculum in an effective way, but can also provide blueprints and best practices for other fields that could benefit from a stronger embrace of empirically supported methods for training students. While the science of learning has been adopted strongly within the health professions, we also note areas for improvement throughout the review, as students (regardless of field of study) are sometimes hesitant to adopt more effective learning strategies compared to less effective ones that they are more familiar with, or they might misunderstand how best to implement the more effective strategies.

We had two major goals for this review. First, to describe and acknowledge the major impact that research on retrieval practice has had on education in the health professions, summarize the efficacy of retrieval practice for learning in this context and highlight how these fields have been using retrieval practice as a learning and study method for the past two decades. Second, to highlight some challenges and future directions for the effective use of retrieval practice in the health professions, many of which are ripe for further empirical study by cognitive psychologists, health professions faculty and even students of the health-professions. We followed Barry et al.’s (2022) six-stage methodology for conducting a state-of-the-art review.

#### Analytical Approach

In Stage 1 (Research Question), we determined our research question and field of inquiry: How have the health professions used retrieval practice to benefit their students? This question and our approach to answering it were guided by our mutual backgrounds as cognitive psychologists and medical education learning specialists (i.e., “MELS”).

In Stage 2 (Timeframe), we determined the period of time that the review would focus on after conducting an overview of the relevant literature. We ultimately chose to organize the review into three important epochs: (1) The (limited) knowledge and use of retrieval practice for learning in the health professions prior to 2010, (2) the embrace and proliferation of retrieval practice by health professions educators from 2010 to 2025 and (3) future directions for retrieval practice in the health professions after 2025.

In Stage 3 (Finalization), we refined the research question and these time frames from our initial assumptions as follows. We initially expected knowledge of retrieval practice in the health professions to be low prior to 2006, when it was highlighted in the cognitive psychology literature by Roediger and Karpicke (2006a). Instead, as our literature review progressed, we recognized that retrieval practice was used heavily in these fields prior to this point, even if it was not labeled as such or its benefits for learning were not fully understood. This status changed greatly by 2010—when it became better understood and identified by name in these fields—so we set our first demarcation to 2010. There was little need for further delineation between 2010 and 2025, as research, understanding and endorsement of retrieval practice in these fields has only grown in that time. Most relevant empirical studies in these domains appeared in the later portion of this period.

In Stage 4 (Search Strategy), we identified articles to include in our review. We first conducted searches for articles containing the phrases “retrieval practice”, “test-enhanced learning” or “testing effect”, in combination with phrases such as “health professions”, “medical school”, “nursing”, etc. We prioritized meta-analyses and systematic reviews, but we also found many individual research papers for some health disciplines which do not yet have relevant review papers. We also needed to identify papers that predated the 2010 demarcation and involved retrieval practice but likely did not include phrases such as “retrieval practice”, “test-enhanced learning” or “the testing effect”. This included searches for articles involving practice-test questions, problem-based learning and using “pimp” or “pump” questions, all of which are forms of retrieval practice that have been stressed in the health professions but are almost never referred to as “retrieval practice” (especially prior to 2010). We also searched for the occurrence of terms related to retrieval practice in websites, handouts and “third party” resources directed at students or faculty of the health professions to determine how widely this topic has been embraced and endorsed in these fields over the years and at present.

We began Stage 5 (Analyses) by reading articles we initially identified in our search, familiarizing ourselves with the literature to date and noting similarities and differences across articles. We also noted gaps in knowledge or understanding, especially if the gaps suggested viable research questions. As noted, this stage included a correction of our initial assumption that retrieval practice was not widely used in the health professions prior to 2006, albeit without such labeling and without knowledge of the benefits of retrieval for learning per se; most early sources viewed forms of retrieval practice such as answering practice test questions for practice and assessment purposes only.

The authors of this review are all cognitive psychologists with unique backgrounds and experiences in research and teaching across psychology and medical education. All currently serve as medical education learning specialists at their respective medical schools. Their shared experience working closely with medical students in individualized instructional settings may have shaped their perspectives when interpreting literature on retrieval practice. For Stage 6 (Reflexivity), we offer the following reflection: although the present review reflects our expertise as cognitive psychologists and as medical–education learning specialists, our interpretation of the state-of-the-art of retrieval practice in the health professions—as well as suggestions for potential new research questions in these domains—is subjective and likely biased by our training and current professional roles. For example, we do not bring to this situation the opinions, experience or expertise of professionals or students of the health professions.

## 2. The Inconsistent Use of Retrieval Practice in the Health Professions (Before 2010)

For the purposes of this review, we will discuss the health professions (i.e., medicine, nursing, dentistry, pharmaceuticals, etc.) as a conglomerate, only noting specific articles, findings or trends from a specific domain when necessary. Though these disciplines all differ from each other in many important ways, in the context of this review they have much in common. As we have already noted, they all require students to learn a high volume of very difficult content in a relatively short amount of time, and they all involve high-stakes exams during the curriculum culminating in high-stakes national licensing exams. There is a clear need for students in all these disciplines to use effective and efficient studying strategies to progress through their curriculum in a timely manner. Obviously, students in these disciplines were studying effectively under these pressures well before the formal recognition of the benefits of retrieval practice for learning by cognitive psychologists in the early 2000s, but how were they doing it, and to what extent did their methods involve retrieval practice?

### 2.1. The Fine Art of “Pimping” Students

There is at least one classical technique in the health professions that clearly emulates retrieval practice as a learning and diagnostic tool, and that is “pimping” (Antonoff & D’Cunha, 2011). This term derives from the German word “Pümpfrage”, which is often translated to English as “pimp questions”, although “pump questions” is a more correct translation (Brancati, 1989). The technique was first described by Harvey in 1628 England and later popularized by Koch in 1889 Germany. This method involves an attending physician (or other person of greater knowledge, such as a senior medical student) rapidly questioning a resident or student of lesser knowledge on complex topics. It is frequently employed during hospital rounds, surgical procedures and academic discussions, and typically occurs in front of peers and patients (Anderson, 2013; Detsky, 2009). By design, the technique is meant to create a high-pressure retrieval situation somewhat like a medical emergency (Brancati, 1989). But it is also meant to encourage learning through negative emotions like fear and intimidation (Gooding et al., 2017; Musselman et al., 2005) in an experience that is embarrassing and borders on traumatic (Jimenez et al., 2024). Some have called for a change in label to this method to avoid an association to pimping/prostitution while still maintaining a negative connotation, including “improper Socratic method” or “malignant questioning” (cf. Nagarur et al., 2019).

Pimping students is often referred to as an “art” by faculty and students in the health professions (Brancati, 1989; Detsky, 2009). Several survey-based studies indicate that faculty and students perceive pimping to be an effective technique for learning and for identifying unknown information (e.g., Abou-Hanna et al., 2021; McEvoy et al., 2019; Rucker et al., 2023; Wear et al., 2005). Given what we currently know about the benefits of retrieval practice for learning, questioning students in this way should enhance their learning and understanding of the information queried and help them identify gaps in their knowledge (cf. Antonoff & D’Cunha, 2011). To our knowledge, however, no study has demonstrated objective improvements in learning from this method (cf. Jimenez et al., 2024; McCarthy & McEvoy, 2015; Reifler, 2015).

Although pimping is still practiced in the health professions today, many articles have questioned the lack of efficacy data and suggested that the negative effects of this practice on students might outweigh any positive effects it might have for their learning. Many faculty and students perceive the practice to be abusive, traumatic and a form of bullying (cf. McCarthy & McEvoy, 2015; Reifler, 2015; Wear et al., 2005). Unironically, Jimenez et al. (2024) hypothesized that the process of pimping could aid students’ learning via the same mechanisms that lead to invasive “flashback” memories in post-traumatic stress disorder. In addition, berating residents and medical students in front of patients and their family members for lacking knowledge might create distrust between health professionals and patients and can stifle communication and deliberation about patient care within teams of health professionals, putting patients at risk (cf. Anderson, 2013).

The process of “pimping” students in the health professions by asking them rapid-fire, difficult questions under social pressure is a pedagogically and ethically questionable practice, but it likely represents an example of using retrieval practice to teach and learn information in the health professions prior to 2010. As with some other forms of retrieval practice utilized in these fields, it was not usually referred to as such before 2010 (in fact, “pimping” is still not often framed as a form of retrieval practice even in 2025; but see Antonoff & D’Cunha, 2011). In contrast to some of the other forms of retrieval practice we discuss in this review, however, the presumed benefits of pimp questions for memory and understanding per se were more often better acknowledged even prior to 2010. That said, we could find no empirical studies that have compared the effects of “pimping” to any other learning modality in the health professions, even lecturing.

Some experts contrast pimping with a classic form of discovery learning known as Socratic questioning. Although both techniques involve teaching by questioning rather than by information delivery, Socratic questioning involves challenging students’ current beliefs to help them recognize their misconceptions and correct their knowledge through critical reflection (Stoddard & O’Dell, 2016; Yudcovitch & Hayes, 2014). Whereas pimping embarrasses or degrades the learner, Socratic questioning requires psychological safety, allowing learners to focus on increasing their knowledge and skill rather than fending off threats to their self-worth (Stoddard & O’Dell, 2016). This method, however, might not produce demonstrable benefits over simple lecturing (e.g., Yudcovitch & Hayes, 2014).

### 2.2. Other Unrealized Forms of Retrieval Practice

Prior to the 2010s, students of the health professions—much like those today—used a combination of information intake (i.e., learning from lectures, textbooks, laboratory activities and treating simulated and actual patients) and forms of retrieval practice (e.g., flashcards, practice test questions, writing summaries, drawing diagrams, working with a study partner, etc.) to learn and practice the content in their curriculum (Thadani et al., 2000). Based on education research and expert guidance at the time, however, it seems that the emphasis was more squarely on information intake as the primary method of learning, but forms of retrieval such as answering practice-test questions were intended only as practice and assessment, not as a learning strategy themselves. For illustrative purposes, let us consider guidance and related research prior to 2010 for how medical students can prepare for the United States Medical Licensing Examination (USMLE) Step 1, the first level of licensing exam that medical students in the United States must pass (typically taken in the second year of medical school).

Problem-based learning (PBL) was a common alternative to the “standard” medical curriculum during this time (Albanese & Mitchell, 1993). Whereas the common approach favored presenting information first and having students apply it later, PBL exposed students to realistic problems and situations before they had learned all the information necessary to do so (e.g., a hypothetical patient presents with given symptoms; students must decide the diagnosis and potential treatment). The intent was to nudge students into an information-seeking mode, where they are responsible for determining what they need to learn for a problem they cannot yet solve (Barrows, 1986). By 2000, medical education articles examining the efficacy of a PBL approach for passing Step 1 focused on its use as a remediation tool for struggling students and found that PBL improved outcomes for those students (e.g., Walters et al., 1999; see also Blake et al., 2000). As with other guidance prior to 2010, PBL articles often discuss related benefits for learning, such as identifying incomplete or incorrect understanding of topics, and building general skills at answering Step 1 questions (e.g., Walters et al., 1999). Although the PBL approach naturally involves students doing many practice questions ahead of Step 1, guidance for this approach did not include the direct benefits of retrieval practice per se on learning.

Similarly, “The Step 1 Method” (Gebremedhin, 2012), a popular study guide for this exam, relies heavily on the use of practice questions as a tool for preparing for the exam. In this case, the primary focus is on using questions for information exposure, repetition, identifying unknown content and practicing and improving test-taking speed and broad question-answering skills. At least in its handbook form (Gebremedhin, 2012), however, this method does not acknowledge the benefits of retrieval practice for learning. Other similar Step 1 courses demonstrated utility for addressing knowledge gaps for students who struggled in the medical curriculum (Thadani et al., 2000), but typically focused on content presentation, review and broad study/test strategies, without any mention of practice questions in a retrieval-practice way (Zhang et al., 2005).

In a research article aimed at MELS and other academic counselors, Coumarbatch et al. (2010) investigated several factors that might predict a student’s likelihood of passing or failing the Step 1 exam on their first attempt. They also suggested several potential interventions for remediation, organized into three levels of severity. Although the specifics of these interventions are sparse, only the intervention designed for the students most at-risk of failing specifically mentions completing practice questions. This aspect of the intervention is called the “3500 Question Challenge”. Presumably, amongst the other things listed (i.e., mandatory academic skills counseling, tutoring, back-to-school reviews, case studies, etc.), these students would have also been expected to complete 3500 practice Step 1 questions. This number might seem daunting to those outside of medical education or the other health professions, but as of this writing (2025), this is the approximate number of questions in each of the two most popular Step 1 question banks (e.g., UWorld and AMBOSS). While completing thousands of practice questions can be beneficial as it promotes retrieval practice, we note that guidelines that focus solely on the number of practice questions to be completed lack nuanced recommendations for how students should be approaching the questions, receiving feedback and reviewing the questions to make the most out of these retrieval practice opportunities.

In contrast, in an early guidebook of study techniques for medical students, Saks et al. (1998) alluded to retrieval practice in a variety of forms within the framework of acquisition, maintenance and proficiency. The authors stressed the importance of cumulative review of concepts using small units of information at regular intervals. For example, one retrieval technique suggested to maintain learning for anatomy was sketching out pathways for cranial nerves from memory, which requires retrieval. Similarly, in learning biochemical pathways, the authors suggest that students draw the pathways from memory to test their knowledge. In the proficiency phase, use of practice questions was encouraged at regular intervals between examinations and well before the student felt ready for the examination. The main objective in their promotion of practice questions was to identify weak topics and ensure that application of knowledge would be successful from initial acquisition to testing. The authors state, “remember that you do not have to reach proficiency to begin questions, as this is a process that can be helpful in deciding what your focus should be” (p. 252). Similarly, in preparation for licensing exams, questions for self-testing were described as an effective way to review information, assess knowledge and prioritize follow-up study. The authors do not use the terms “retrieval practice” or “testing effect” but consistently support that recall is highest when regular review of information that is organized in a meaningful way is completed and use of practice questions early and often are stressed throughout their text. Importantly, we must acknowledge that this guidebook identified and anticipated many of the benefits of retrieval practice for studying and learning—based on the authors’ experience advising medical students for academic success—that are now taken as fact after decades’ worth of research in cognitive and educational psychology.

In summary, the dominant educational emphasis in the health professions prior to the 2010s was on content exposure rather than retrieval as a core learning mechanism. Health professions students typically focused on a combination of information intake—via lectures, textbooks and clinical experiences—but practice questions were reserved for assessment of progress. Problem-based learning (PBL) was a notable alternative approach that encouraged active information seeking but also lacked explicit recognition of the learning benefits of retrieval practice. Popular study guides and courses that prepared students for licensing exams similarly emphasized question volume without describing the benefits of engaging with the questions (a tendency that persists in many fields besides the health professions; cf. Hartwig & Dunlosky, 2012). An exception was the early work of Saks et al. (1998), who, without using terms like “retrieval practice,” described study methods that strongly aligned with its principles—such as recalling information from memory, cumulative review and early use of practice questions to guide study.

### 2.3. Research on “The Testing Effect” Begins

Besides guidance in books, curriculum and study guides, there are also examples of health professions research examining the effects of retrieval practice on learning prior to its formal recognition and labeling in cognitive psychology (i.e., Roediger & Karpicke, 2006a). For example, Van Hoof et al.’s (2023) review of retrieval practice studies in nursing includes several studies that predate this time point: two from 2001 and one from 2006. Trumble et al.’s (2024) review includes some studies by Logan et al. (1975), who examined massed practice (studying a topic in one large “block” of time) versus distributed or spaced practice (studying a topic repeatedly or in sections spaced over time, often with the study of other content interspersed) in the acquisition of physical dentistry skills. Although distributed or spaced study does not explicitly involve retrieval practice, it likely benefits from some of the same mechanisms as both require the reactivation of information at a delay.

Shortly after the modern, formal recognition of the benefits of retrieval practice for learning in cognitive psychology (i.e., by Roediger & Karpicke, 2006a), several articles appeared by Larsen, Butler, Roediger and others (e.g., Larsen et al., 2008, 2009, 2013, 2015) indicating a clear partnership of cognitive psychology and medical education researchers examining retrieval practice in a medical education context. In the first such article, Larsen et al. (2008), suggested that, “If test-enhanced learning were to be implemented in medical education, the focus of testing and assessment might shift significantly. Tests would no longer be considered neutral tools of measurement, but rather active instruments to aid in the acquisition and retention of knowledge.” (p. 963). Although brief, their paper and its framing clearly suggest that retrieval practice per se was not yet explicitly endorsed in medical education (see also Larsen et al., 2009). Presumably, it was not yet recognized in the other health professions either. To Larsen et al.’s credit, when they suggested that tests would become “active instruments to aid in the acquisition and retention of [medical] knowledge.”, they accurately predicted what would soon become the norm in the health sciences less than fifteen years later: students using various forms of retrieval practice not just as a form of assessment but also as a form of learning.

## 3. Recognition, Verification, and Adoption of Retrieval Practice in the Health Professions (2010–2025)

By the 2010s, knowledge of the benefits of retrieval practice had clearly reached the health professions. Initiated by studies and papers involving both cognitive psychology researchers and health professions researchers (e.g., Larsen et al., 2008, 2009), the historical record indicates a rapid proliferation of papers examining the effects of retrieval practice in authentic, health profession contexts (especially in the last decade). For example, Deng et al. (2015) found that medical students frequently use self-initiated retrieval practice (e.g., use of digital flashcard products), with or without spaced or distributed repetition, to prepare for the Step 1 licensing exam, and that such retrieval practice enhances learning and test performance. Metz et al. (2021) found that spaced or distributed retrieval practice improved year one dental students’ learning outcomes. Studies by Giuliodori et al. (2008) and Kleinsorgen et al. (2018) indicate that retrieval practice aids the learning of veterinary medicine content. Pre-lecture quizzes can enhance students’ memory for neuroanatomy and physiology terminology and concepts (Pan et al., 2019). Articles also support the use of retrieval practice while studying for the Medical College Admission Test (MCAT), a standardized examination for prospective medical students commonly used in the United States and in some other countries (e.g., Steed & Kadavakollu, 2019; Swan Sein et al., 2020).

Several review papers summarize findings from studies within a specific health domain (e.g., just nursing), across multiple health domains, or along with studies from many other diverse fields (see Table 1 for some examples). Importantly, most studies and meta-analyses demonstrate positive benefits for retrieval practice in health professions education. Green et al.’s (2018) review across the health professions found robust benefits of retrieval practice for learning, including effects that extend beyond course examinations to clinical applications. Trumble et al. (2024) also found that retrieval practice was effective at improving academic performance in the health professions in the majority of the studies they reviewed. Thompson and Hughes (2023) argued that retrieval practice helps the learning of radiology content, but their review only identified eight articles that met criteria, with just five finding a positive benefit of retrieval practice. Goldman et al. (2024) found that the use of Anki flashcard software (presumably, Version 2.0 or 2.1 given the timeline) was positively associated with exam outcomes in the health professions. Wu et al. (2021) identified several factors positively associated with medical students’ scores on the Step 1 exam (before that exam changed to pass/fail in 2022), including some that reflect retrieval practice such as greater use of flashcards and practice questions during study.

Most empirical articles examining retrieval practice in a health professions context were published between the years 2010 and 2025. Furthermore, as indicated by several of the reviews in Table 1, most of these articles were published at the more recent end of that range. This pattern suggests that the knowledge and empirical examination of retrieval practice in the health professions only continues to grow. That said, there are some articles in the reviews in Table 1 that appeared before 2010, and even before the publication of the work by Roediger and Karpicke (2006a). Per our discussion of the “inconsistent” use of retrieval practice in the health professions prior to 2010, these data points not only support our earlier suggestion that students of the health professions were using various forms of retrieval practice to aid their learning prior to 2010 (and 2006), but also that researchers in these domains were studying it in various forms, albeit without the more common labels for the effect that would follow in cognitive psychology such as “the testing effect”.

Health professions research involving the effects of retrieval practice also appears in domain-general reviews of the effect (e.g., Moreira et al., 2019). For example, Agarwal et al. (2021) included several papers examining the topic in medicine in their review. Those authors noted that retrieval-practice research in the medical context is often very applied and authentic and that it typically uses rephrased questions rather than repetitions of old questions as is common in some laboratory studies and in some other fields. Of importance here, some cognitive psychology studies have failed to find “far transfer effects” of retrieval practice such that the benefits of retrieval for learning are strongest when the retrieval practice tests the same information as the final criterion test but is weaker or absent when the information tested at both times is less closely related (cf. Wooldridge et al., 2014; Yeo & Fazio, 2019). As well, many such studies in the medical context involved designs where retrieval practice was compared to active, “non-retrieval” comparisons, not simply compared to students not using any form of restudying at all. These are all good markers of quality applied work but also help to ensure that we can trust that retrieval practice is helpful for learning in the health professions context.

### 3.1. Adoption in Formal Training or Curriculum

It is difficult to fully assess the extent to which retrieval practice is formally endorsed as an effective learning and study method in the health professions. For example, a faculty member or learning specialist might advise a struggling student to use retrieval practice and to complete practice questions while studying, but there would be no public record of that endorsement. Nevertheless, some evidence exists for the formal recommendation of retrieval practice in the health professions across published empirical papers indicating its efficacy, in opinion pieces written by faculty or students, on the websites of schools of the health professions, in supporting documents from third-party learning resources and in online content written by past and present students of the health professions. Table 2 includes several such examples but is certainly not an exhaustive list. Although most of these resources are aimed at the individual student/learner who is likely engaging in self-regulated study on their own, some of these resources are aimed at faculty, who can affect the study behaviors of multiple students at once through course and assignment design or via explicit instruction to students about the utility of retrieval practice for learning.

Larsen et al. (2008) proposed that retrieval practice aligns with the use of assessment to improve medical education and provides a valuable tool to help students to learn and to retain essential knowledge. Thus, some health professions faculty have incorporated retrieval practice through frequent, low-stakes testing (cf. Swan Sein et al., 2021b). Deng et al. (2015) also encouraged medical schools to explore these methods and to research optimal implementation strategies. As the content in Table 1 and Table 2 indicate, numerous empirical and editorial articles support including retrieval practice in health professions education. In addition, some schools explicitly discuss and recommend retrieval practice on their websites and in other resources. These resources, which have greatly expanded since 2010, reference key cognitive psychology concepts such as retrieval practice, spaced retrieval and metacognition, citing studies from cognitive and educational psychology as well as health professions research to back those assertions.

### 3.2. Adoption in the “Parallel Curriculum”

Table 2 also indicates that we can find the explicit explanation and endorsement of retrieval practice in supporting documents created for the third-party learning resources commonly used in the health professions (e.g., Anki, n.d.; AMBOSS, n.d.; UWorld, 2021), and in blogs and social media posts written by students and others in the health professions. As noted earlier, such writing about how to use those resources is part of the “parallel curriculum” in the health professions (Barrison et al., 2024). Many modern educational resources such as flashcard software have adopted retrieval practice and other findings from cognitive psychology into their design. Students of the health professions also obtain “unofficial” information from their peers, both in-person and online. In their summary of how heavily medical students (and presumably students of the other health professions as well) rely on online forums such as Reddit for study advice, Ronner and Linkowski (2020) suggested that, “On the basis of forum recommendations and advice from their peers, students couple this type of flashcard schedule with a number of commercial Step-prep resources … to build a rigorous, months-long study schedule devoid of any input from a brick-and-mortar medical school. … [T]his is increasingly the new normal in preclinical medical education.” (p. 1330). As such, it is paramount that students receive tips and information embedded into these resources that clearly and accurately describe the benefits and rationale for effective study techniques such as retrieval practice or spaced repetition, as these resources—and advice from their peers—might be the primary source(s) from which they learn about such techniques.

## 4. Broadening and Strengthening the Use of Retrieval Practice in the Health Professions (2025 and Beyond)

Retrieval practice is clearly effective at increasing learning and exam performance in the health professions (cf. the reviews in Table 1), and students in these fields are using various forms of retrieval practice as they study. But that does not mean that the issue is settled; there are still barriers to students of the health professions using retrieval practice and using it effectively and efficiently. Accordingly, we have organized the final section of this review into three subsections, reviewing some common barriers to the use of retrieval practice by students of the health professions, interventions that could increase its use in these fields and factors that can make retrieval practice more efficient or more effective. Any of the following topics would benefit from further empirical research by cognitive psychologists and health education researchers.

### 4.1. Barriers to Retrieval Practice

Despite the numerous benefits of retrieval practice for learning, some students are hesitant to engage in forms of retrieval during the learning process. Other factors simply make it difficult or less appealing for students to do so. In this section, we review some major reasons why students in the health professions might avoid retrieval practice.

#### 4.1.1. Lack of Knowledge About Retrieval Practice

Despite the surge in research related to retrieval practice in the past 15 years, this information does not seem to have reached students and educators more broadly (but see Table 2). Indeed, when asked about the frequency with which they use various strategies, undergraduate students report re-reading at a much higher rate than retrieval practice (Blasiman et al., 2017). Those students who engage in retrieval practice often do so for assessment purposes and not as a learning strategy (Hartwig & Dunlosky, 2012). This may be partially due to educators’ inadequate knowledge of retrieval practice as an effective learning strategy as well. Halamish (2018) asked educators to choose the better learning strategy amongst two scenarios, and retrieval practice was only endorsed 30% of the time. More recently, however, Witherby et al. (2025) found that retrieval practice was rated as the most effective strategy by undergraduate psychology students, as well as by educators and parents in the general population, so knowledge of the effect might be increasing. We should note, however, that such studies typically describe behaviors in everyday terms, which respondents either endorse as using (or not), or rate the methods in terms of their presumed efficacy for learning. Such studies rarely, if ever, use cognitive psychology terms such as “retrieval practice” when describing these behaviors for rating, which could dissuade broader spread of the topic. In contrast, the resources listed in Table 2 and others like them typically do use cognitive psychology terms and explanations for their efficacy, perhaps under the assumption that students of the health professions can understand such ideas better or will be more likely to engage in self-testing when framed in a cognitive psychology context than would elementary school children or their parents.

#### 4.1.2. Choosing Less-Effective Learning Strategies

By and large, data from the health professions indicates that students in these areas mimic those in traditional higher education settings: even when students are aware of the benefits of retrieval practice, they tend to choose other less-effective strategies for self-regulated learning (Corazza et al., 2023; Klein et al., 2023; Sheehy et al., 2024). There is far less data to indicate exactly how students engage in retrieval practice during self-regulated learning, and this is an area ripe for investigation. In one recent study, Sheehy et al. (2024) found that students do not always use retrieval practice in the most effective way. For example, students might choose to look up the answer to a question if they are unsure or have limited knowledge in an area or choose to only engage in additional retrieval for topics that they missed. Both strategies indicate a limited awareness of how retrieval practice is most effective, and result in limited benefits for retrieval practice.

As well, some students seem to be aware but untrusting of the benefits of retrieval practice for learning. While the research is somewhat limited, students in both medical (Furness et al., 2024) and dental education (McAndrew et al., 2015) report choosing re-reading and other less effective strategies more often than retrieval practice. Some of this hesitation might be due to the sources of information that students use when determining how to study. For example, in discussing medical school forums on Reddit, Ronner and Linkowski (2020) noted that students on these forums, “regularly receive and reply to direct messages from users on Reddit asking for the type of advice that a learning professional or study counselor hired by an institution should be providing. The fact that struggling students may be more willing to seek academic help and mentorship from an anonymous online peer than from an educator at their own institution says something meaningful about the alienation students feel from those who are tasked with teaching them.” (p. 1330). Ironically, students might interpret their peers’ infrequent endorsement of retrieval practice (compared to faculty and learning specialists) as an indicator that it is not an effective strategy.

That said, Kann et al. (2024) recently analyzed the content of the 500 top-ranked r/Step 1 subreddit posts between 26 January 2022 and 10 April 2023. Of the materials most frequently mentioned, the top two were “NBME [National Board of Medical Examiners] materials” (51.40%) and UWorld (40.40%). UWorld is a question bank, and “NBME materials” likely included self-assessment and practice exams that students can take while studying for the Step 1 exam. This may be an indication that students are using retrieval strategies without fully understanding why they are beneficial, which has the potential to lead to “lethal mutations” in the efficacy of the method (Bagley, 2020) due to misperceptions about how retrieval practice “should feel” or the immediate outcomes associated with it. For example, people tend to assume that subjective ease or fluency is an indicator of successful performance, such as effective learning, even when it is not (e.g., Kelley & Lindsay, 1993; Koriat, 2008). In some cases, effortful processing (including effortful retrieval) is associated with better learning or future performance (Benjamin et al., 1998; Serra & Dunlosky, 2005). Students likely need to change how they incorporate experienced fluency into self-evaluations of learning (Briñol et al., 2006).

At the same time, not all recommendations and practices in the health professions are as clearly based on current and accurate empirical knowledge regarding effective studying or learning. While faculty are experts in their respective health professions, they may not be well-versed in cognitive psychology principles that optimize student learning. Consequently, some guidance for faculty continues to promote ineffective methods such as the consideration of learning styles in teaching (e.g., Friedlander et al., 2011) while overlooking empirically supported strategies like retrieval practice and spaced repetition (Augustin, 2014; Selva-Rodriguez & Sandars, 2023; Swan Sein et al., 2021a). Recognizing this gap, the Liaison Committee on Medical Education (LCME) requires structured academic advising that integrates faculty, course directors and other student-support services (see LCME Accreditation Standard 11.1). In response, medical schools have increasingly hired learning experts from fields like cognitive psychology, education and neuroscience to develop and implement evidence-based learning strategies (Rashid et al., 2024). These MELS serve as a bridge between research on learning science and practical application in medical training, ensuring that faculty and students benefit from proven instructional techniques without requiring all educators to master these principles. By leveraging the expertise of MELS, medical schools can provide faculty (and students) with accurate, research-backed resources while allowing them to focus on their primary role: teaching and mentoring future healthcare professionals.

#### 4.1.3. Misleading Experiences of Retrieval Practice

Students might remain or become hesitant to use retrieval practice while studying because they have previously relied on some forms of retrieval practice that were not appropriate for their learning goals, leading to unimpressive results on assessments. Such experiences can lead students to assume that all forms of retrieval practice are inefficient. For example, students of the health professions make heavy use of flashcard software such as Anki to study for high-stakes exams (e.g., Barrison et al., 2024; Deng et al., 2015). Although flashcard software produces impressive memorization, the quality of learning and future test performance is very dependent on the quality of the flashcards studied and how closely that learning compares to the actual exam(s). Put differently, a student who is effectively memorizing low-level facts via flashcard software might nevertheless struggle on a course or licensing exam where answering questions correctly requires a much more advanced level of comprehension. That student might then assume that all forms of retrieval practice are inefficient for learning, whereas the problem was instead with the type of retrieval practice they utilized. Rather, for students to most effectively transfer their knowledge from retrieval practice to course or licensing exams, it is best if the practice matches the type of processing required on the exam (Barnett & Ceci, 2002; Morris et al., 1977). Students might also wait to use retrieval as a practice assessment shortly before their summative exam to determine their performance. Because students are waiting too long to start using practice questions, they are not maximizing the learning utility of retrieval practice and may not recognize its benefits (cf. Saks et al., 1998). A student in these situations might also assume that all forms of retrieval practice are inefficient and return to less-effective study methods which do not produce a discordant experience (cf. McAndrew et al., 2015; see also Karpicke et al., 2009).

Students often judge their own learning based on the speed and ease (fluency) with which they can retrieve information from memory. In knowledge-based tasks, faster responses are more likely to be correct (Hertwig et al., 2008; Robinson et al., 1997) and considering response speed improves the accuracy of students’ judgments of their own knowledge (Koriat & Ackerman, 2010; Serra & Dunlosky, 2005). The experience of fluency, however, can be misleading: the repeated exposure to potential answers also increases response speed and confidence, even if the answers retrieved quickly are incorrect (Kelley & Lindsay, 1993; Koriat, 2008). In a broader sense, attempting retrieval in various forms is often experientially more effortful than many other forms of studying or practice such as re-reading text or re-watching videos that are less likely to produce long-term changes in students’ learning. For this reason, students might favor low-return study and restudy techniques over forms of retrieval practice because the former feel easier to engage in.

#### 4.1.4. Negative Beliefs About Testing and Errors

Some students hesitate to use practice questions or other forms of retrieval practice for studying, practice, or self-assessment because they hold negative beliefs about making errors. (cf. Chouvalova et al., 2024; see also Carpenter et al., 2020a, 2020b; Dresel et al., 2013; Pan et al., 2020; Tulis et al., 2017). A well-known topic in psychology and in the health professions is mindset: while a growth mindset refers to the belief that personal traits such as intellectual abilities can be improved over time, a fixed mindset is the belief that these traits are static and cannot be changed (Yeager & Dweck, 2012). Recent research with undergraduate biology majors indicates that some students possess positive beliefs about making errors during learning (i.e., we benefit from making errors during the learning process), but others possess negative beliefs about such errors (i.e., errors are harmful and to be avoided). Although a growth mindset might be necessary for students in the health professions to succeed academically, it is likely not enough.

A further implication of research on error avoidance (Chouvalova et al., 2024; see also Dresel et al., 2013; Tulis et al., 2017) is that students might be hesitant to use practice questions or other forms of retrieval during study because they worry that being unable to retrieve information or answer questions correctly will signal that they do not know the information, or might not ever be able to learn it (a form of fixed mindset). Such concerns are likely related to students’ experience of “imposter syndrome”, a pattern of behavior in which people doubt their abilities or fear being exposed as a fraud, even if they have adequate abilities in a domain (such as medicine; see Mullangi & Jagsi, 2019). Students who are high on imposter syndrome are less likely to ask for help and do not want to be “outed” as an imposter (Chen & Son, 2024). Related, many medical students experience forms of academic shame around poor performance and other metrics which suggest they are not performing as well as their peers (and leads to reduced help-seeking behaviors; see Coudret, 2020). Students of the health professions who experience imposter syndrome or feelings of academic shame might avoid completing practice questions or other forms of retrieval practice to avoid such feelings or to avoid being “outed” as an imposter, sacrificing the benefits of retrieval practice to spare their egos.

Other than Coudret (2020), who conducted interviews with medical students about the sources and consequences of their feelings of academic shame, the empirical work above regarding negative beliefs about errors (e.g., Chouvalova et al., 2024) and imposter syndrome (e.g., Chen & Son, 2024) mostly involves undergraduate students. Although we would expect that the same patterns might occur with students of the health professions (i.e., imposter syndrome seems common in medical students; see Mullangi & Jagsi, 2019), these topics need exploration with these specific populations. We know of no research that has examined error avoidance per se in the health professions.

### 4.2. Addressing Barriers to the Use of Retrieval Practice

Despite the barriers that may deter health profession students from using retrieval practice, strategies exist to increase its adoption. Two recent reviews (Carpenter, 2023; Rivers, 2021) offer recommendations for promoting its use in students broadly.

#### 4.2.1. Improving Knowledge About Retrieval Practice

Educating students about retrieval practice and its benefits could increase students’ use of retrieval practice. Table 2 lists resources aimed at informing health profession students, faculty and tutors. However, merely providing information does not usually lead to significant behavioral change (Ariel & Karpicke, 2018; Carpenter, 2023; Morehead et al., 2016; Simone et al., 2023). Rivers (2021) acknowledged these challenges but also highlighted studies demonstrating benefits from explicit instruction (e.g., Einstein et al., 2012; Lineweaver et al., 2019; McCabe, 2011). Health profession students frequently use retrieval practice but often value peer advice over faculty guidance (Ronner & Linkowski, 2020). Thus, engaging senior students to promote its use among peers can be effective (Jodrell, 2010). Additionally, educating students on efficient study techniques can enhance their role as peer learning specialists or tutors. Creating a supportive environment with faculty and administrators may further encourage adoption (Zumbrunn et al., 2014).

#### 4.2.2. Improving Experiences with Retrieval Practice

Another strategy involves providing experience with retrieval practice. Students often revert to less effective methods despite prior use (Hui et al., 2021, 2022; Kirk-Johnson et al., 2019). Because retrieval practice feels difficult, students may mistakenly perceive other more fluent methods as being more effective (Kelley & Lindsay, 1993; Koriat, 2008). Carpenter (2023) emphasized the importance of teaching students that effortful retrieval is beneficial, a form of “desirable difficulties” (Bjork & Bjork, 2023; Carpenter et al., 2020a, 2020b). Explicit feedback can further help students recognize the effectiveness of retrieval practice, increasing subsequent use (Carpenter et al., 2017; Hui et al., 2021, 2022).

Educational technology can also support retrieval practice (Carpenter, 2023). Many digital tools in the health professions offer practice-test questions, performance feedback and analytics that highlight weaknesses. Such tools not only educate students but also reinforce the use of retrieval practice through experience and feedback (cf. Table 2).

#### 4.2.3. Addressing Negative Beliefs About Testing and Errors

Chouvalova et al.’s (2024) research with undergraduate biology majors indicates that, although having a fixed mindset did not relate to students’ study strategy selection or use (presumably because they did not think that trying different strategies would matter for their learning), the effect of a growth mindset on students’ achievement was mediated by their beliefs about errors. Specifically, holding positive beliefs about errors led students to choose more effective study strategies, such as spaced study and retrieval. As discussed above, more research is needed to better understand how error avoidance and imposter syndrome affect study strategy choices for health profession students, and to develop interventions to address those negative beliefs. Potentially effective interventions might target students’ negative beliefs about errors (Chouvalova et al., 2024) and increase their understanding of the positive benefits of effortful processing and retrieval (Carpenter, 2023). Importantly, at least one study has found that the benefits of retrieval practice for learning might be larger for students with a low mastery orientation versus a high mastery orientation (Endres & Eitel, 2024, Experiment 1). As well, the effects can be enhanced with explicit rewards that incentivize learning (Endres & Eitel, 2024, Experiment 2).

#### 4.2.4. Improved Self-Regulated Learning Strategies

Students often choose less-effective strategies for self-regulated learning, even when they are aware of the benefits of retrieval practice (Corazza et al., 2023; Klein et al., 2023; Sheehy et al., 2024). More research is needed to better understand how health profession students engage in retrieval practice during self-regulated learning and how to improve the use of retrieval practice during self-regulated learning. Combining interventions may be necessary for widespread adoption. Several academic programs centered on retrieval practice, such as The Study Smart Program (Biwer et al., 2023), The Start and Stick to Desirable Difficulties framework (de Bruin et al., 2023) and The Knowledge, Belief, Commitment, and Planning framework (McDaniel & Einstein, 2020), all integrate multiple strategies. Of note, both The Study Smart Program and Desirable Difficulties framework have successfully improved performance in health profession students (Biwer et al., 2023).

### 4.3. Enhancing the Efficacy of Retrieval Practice

There are many opportunities for further improving retention of health professions material across curricula, and arguably it is critical within the health professions where students are preparing for large comprehensive or licensing exams and—arguably—these students’ “true final exam” is their clinical practice. Within courses, however, students should be provided with many low-stakes retrieval attempts that can be used formatively to improve learning in addition to improving metacognition and realization of the utility of retrieval practice. In some universities, formative assessments are implemented (e.g., Arja et al., 2018; Lakhtakia et al., 2022), but the degree to which these assessments are used as retrieval practice (as opposed to providing students with metacognitive awareness or to guide future study) is unclear. In professions where students are being prepared for patient care, using evidence-based learning strategies becomes a moral imperative.

When students first enter the curriculum in the health professions, they have a wide variety of prior knowledge. Because retrieval practice is more effective when retrieval is successful (Chan et al., 2024), some students (i.e., those who understand a topic less are less likely to be able to retrieve information) may need more time with explicit instruction and content review before they engage in retrieval practice (Kalyuga, 2007). Furthermore, students may start engaging with retrieval practice at a factual level to increase retention and understanding. However, health professions education, by its nature, necessitates a considerable amount of transfer of knowledge from the classroom to applied settings (Patel & Cranton, 1983; see Kaminske et al., 2020 for discussion). Often, exams within courses involve case vignettes or patient confederates to which students must apply their factual understanding. While students certainly need to acquire a vast amount of factual knowledge to be successful, utilizing retrieval practice solely for low-level recall of facts may not lead to large performance gains on applied exams. Although data indicates that such flashcard use is positively related to performance on such exams (e.g., Deng et al., 2015; Wu et al., 2021), it is incorrect to assume that flashcards alone can produce sufficient learning or comprehension for excelling on these exams. This is particularly true when students utilize flashcard-based programs where they repeatedly see the same factual items spaced out over time. The effectiveness of these flashcard systems depends on how students utilize them. Often, students will only keep a card in a deck until they have answered it correctly once, dropping it from further retrieval attempts, which is far less effective than completing multiple retrievals (Karpicke & Roediger, 2007). Students might also become so familiar with their deck of cards that they stop engaging in full retrieval of the answers, which is typical of covert retrieval (Sumeracki & Castillo, 2022).

One of the major benefits of flashcard software programs over physical flashcards is that they are often designed to create expanding, spaced retrieval practice. However, it is unclear exactly how the algorithms for many of these programs are designed, whether they are designed to maximize learning, and whether students use them the way they are intended. A notable exception is Anki, an open source digital flashcard program used by students in various disciplines to study for exams, including students in the health professions (Deng et al., 2015). Anki’s documentation includes discussion of the benefits of both retrieval practice and expanding retrieval practice (Anki, n.d.). Both factors are key to its design and efficacy. Early algorithms used in Anki software relied on seeing new information frequently and then, based on successful retrieval, expanding intervals (e.g., every three days, every week, every two weeks). This model relied on students feeding information into the algorithm through rating the ease in which they were able to recall information. As noted earlier, because students’ metacognition is often misaligned with their performance, less effective retrieval schedules may often be implemented. In addition, when students build up many new cards, it limits their ability to continue the repetition of older cards even if they have not yet mastered that content. For example, a popular Anki card deck designed for the USMLE Step 1 exam, “ANKING”, has over 30,000 cards that some students try to master in an 18 month to 2-year period. Attempting to master so many flashcards is extremely time-consuming and can limit students’ ability to engage in more effective study behaviors including attending lectures and answering vignette-style practice questions. Newer algorithms have emerged for Anki, including the Free Spaced Repetition Scheduler algorithm that uses machine learning to learn the user’s memory patterns and allows greater options for delaying or speeding up reviews (Ye et al., 2022). Examining ideal schedules and algorithms for such programs is a much-needed area of further research and refinement.

We should note that most of our discussion about the efficacy of retrieval practice has focused on student self-directed learning. Retrieval practice, however, can and should be incorporated into health professions courses. Indeed, institutions have incorporated retrieval practice to varying degrees. In one study, Azzam and Easteal (2021) found that implementing retrieval practice at the beginning of class significantly improved final exam performance in Gross Anatomy. As suggested in Table 2, many university webpages discuss the use of retrieval practice as a curricular tool. However, it is unclear both from the published literature and from these webpages the degree to which retrieval practice is being implemented within and across health professions curriculum and this content likely varies considerably across instructors and institutions.

Admittedly, our review has primarily focused on forms of retrieval practice that involved the recall of explicit information from semantic or episodic memory (e.g., using flashcard software or completing practice questions during study), usually in the service of preparing to answer test questions that also test explicit semantic or episodic memory (e.g., multiple-choice medical licensing exam questions). But either retrieval practice or the final test could involve tests of procedural learning, which reflects our implicit level of skill acquisition (e.g., a medical student’s ability to physically insert a urinary catheter, rather than their explicit knowledge of how to do so). To date, however, very few studies in the domains of cognitive psychology or educational psychology have examined the effects of retrieval practice of any kind on skills learning, or of procedural practice on explicit or implicit learning. Even fewer have done so in the context of health professions, but those studies have found positive results. For example, Kromann et al. (2009, 2010) found that explicitly testing a group of medical students on their resuscitation skills just after completing a training course led to higher performance on a final test, compared to a group who completed the same course content but without the intervening test. Although the final test was explicit in nature, the retrieval practice involved implicit recall (performing the task). Conversely, Ndoja et al. (2022) found that explicitly retrieving procedural information–specifically, writing down the steps of an orthopedic surgery–reduced forgetting from pretest to posttest compared to a group that restudied the steps. In this case, although the retrieval practice involved the explicit retrieval (writing down) of the steps involved in the surgery, the final test involved students actually performing the procedure. Such findings indicate that the learning of procedural information in the context of the health professions can benefit from retrieval practice (whether explicit or implicit in nature), and that procedural practice can have similar benefits for learning as does explicit retrieval practice. To date, however, these topics are under-studied in these contexts despite the importance of procedural skills for many aspects of the health professions. An exception has long been surgical procedures, where learning is often done by repetitive practice or simulation (cf. Anton et al., 2019; Rosenthal et al., 2010; Scott et al., 2011). That said, whether or not practicing procedural skills involves “retrieval” is a debate that extends far beyond the boundaries of this review. It is clear that people can improve their skills with practice, but explicit retrieval does not need to occur for this to happen, as people without episodic memory can improve their procedural learning with practice (Squire, 2009).

## 5. Summary and Conclusions

In this review, we have attempted to summarize and describe the major impact that research on retrieval practice has had on education in the health professions. Research on retrieval practice within cognitive psychology experienced a resurgence in the early 2000s (i.e., Roediger & Karpicke, 2006a) and quickly found its way into medical education (e.g., Larsen et al., 2008, 2009) and the health professions more generally. From there, health profession researchers and educators were quick to embrace retrieval practice, with most empirical studies of the topic in these contexts occurring within the last decade or so. It is difficult, however, to assess the full extent to which retrieval practice is used within health professions education for at least two reasons. First, learners and researchers have likely been using and studying retrieval practice in various forms, using different labels (e.g., distributed or spaced practice, “pimping”, etc.). Second, health profession students and trainees receive advice and instruction about learning strategies from several different sources including formal curriculum, MELS, third-party resources and—increasingly—online recommendations from peers (e.g., Reddit, TikTok and YouTube). Thus, while there appears to be a broad adoption of retrieval practice in health professions education, the advice and nuance on how to use retrieval practice is somewhat fractured.

In many ways, the health professions are an ideal setting for adopting retrieval practice. Students and trainees must learn an ever-increasing amount of information, take high-stakes licensing examinations, are likely high-performing students, their training involves a mix of lecture-based instruction and hands-on clinical experience, and involves heavy use of third-party online resources that rely strongly on question-banks. All of these factors contribute to an environment and culture where students are highly incentivized to use flashcards and question banks to make their learning as efficient as possible. Despite the widespread use of retrieval practice, there are still barriers to students using it effectively. First, students may be unaware of the benefits of retrieval practice as many seek information about how to study through unvetted social media sources. Second, students’ previous experiences with retrieval practice may dissuade them from further implementing retrieval practice. They may have either used low-effort forms of retrieval practice and been unimpressed with the results (e.g., overly relying on flashcards), or were discouraged by experiencing low levels of fluency during retrieval practice (see Karpicke, 2009). Finally, negative beliefs about errors could lead students to avoid using retrieval practice altogether (Chouvalova et al., 2024).

Retrieval practice has clear benefits for health professions education where the transfer of learning to practice is both clear and important, yet there remains limited research to date on how students use retrieval practice for self-regulated learning. While institutions have increased their use of active learning, retrieval practice as an explicitly used learning tool is still limited. Despite the broad awareness and interest in retrieval practice, it remains under-researched in health professions education. In addition, we could make greater efforts to train faculty and staff in the health professions to allow them to better understand how and why retrieval practice benefits learning. While students are the primary users of retrieval-based strategies, instructors and academic support staff play a crucial role in shaping educational environments and reinforcing effective learning techniques. Many faculty members may be unaware of the robust evidence supporting retrieval practice or how to implement it effectively in both classroom and clinical settings. Providing targeted professional development and training could help close this gap, ensuring that educators are well-equipped to guide students in applying retrieval-based strategies in more purposeful and informed ways.

## Figures and Tables

**Table 1:** Examples of recent reviews on the efficacy of retrieval practice for learning that include the health professions.

Discipline	Review Type	Authors/Year	Studies	Study Years
Medicine Only	Systematic	(Wu et al., 2021)	29	2005–2019
Nursing Only	Scoping	(Van Hoof et al., 2023)	25	2001–2021
Radiology Only	Systematic	(Thompson & Hughes, 2023)	8	2014–2022
Health Professions	Systematic	(Green et al., 2018)	19	2009–2016
	Scoping	(Barrison et al., 2024)	64	2011–2024
	Systematic	(Goldman et al., 2024)	8	2015–2022
	Systematic	(Trumble et al., 2024)	63	1975–2022
Health and Others	Meta-Analysis	(Adesope et al., 2017)	3 *	2009–2010 *
	Meta-Analysis	(Pan & Rickard, 2018)	7 *	2009–2013 *
	Systematic	(Moreira et al., 2019)	4 *	2007–2015 *
	Systematic	(Agarwal et al., 2021)	10 *	2009–2017 *
	Systematic	(Rivers, 2021)	6 *	2015–2019 *
	Meta-Analysis	(Yang et al., 2021)	93 *	1968–2019 *

* Note that, for the six reviews that included health profession studies along with non-health profession studies, we have only included information (i.e., number of studies and years of those studies) for those studies that were directly relevant to the health professions.

**Table 2:** Example journal, university, and third-party resources explicitly endorsing the use of retrieval practice for learning in the health professions.

Type	Discipline	Source	Title
Journal Article	Anesthesiology	Weidman and Baker (2015), *Anesthesia & Analgesia*	“The Cognitive Science of Learning: Concepts and Strategies for the Educator and Learner”
	Medicine	Cutting and Saks (2012), *Medical Teacher*	“Twelve tips for utilizing principles of learning to support medical education”
	Medicine	Swan Sein et al. (2021a), *Medical Teacher*	“Twelve tips on guiding preparation for both high-stakes exams and long-term learning”
	Medicine	Ahmed et al. (2021), *Medical Teacher*	“Optimizing preclinical learning with retrieval practice: A call to action”
	Medicine	Coppola et al. (2023), *Academic Medicine*	“Determining Root Causes of Poor Academic Performance to Provide Wraparound Support for Preclerkship Medical Students”
University Resource	Medicine	(Loma Linda School of Medicine, 2021)	“The Science (and Illusions) of Learning: Instructional Design Tips for Teachers to Enhance Learners’ Long-term Memory Retention”
	Medicine	(The Robert Larner College of Medicine at University of Vermont, n.d.)	“The Science of Learning. Learning Theory: Retrieval Practice”
	Nursing	(UConn School of Nursing—University of Connecticut, n.d.)	“Learning Science Strategies Series, Module 3: Retrieval Practice”
Third-Party Resource	Multiple Fields	(Anki, n.d.)	Anki Manual: “Background”
	Medicine	(AMBOSS, n.d.)	“Internal Medicine (ABIM) Board Review”
	Nursing	(UWorld, 2021)	“Practice Questions: More Important than Ever for NCLEX Success”
	Medicine	(Lecturio, 2024)	“Active Recall and Retrieval Practice in Medical Education”
	Medicine	(Student Doctor Network, 2018)	“Gain a Deeper Understanding with the Power of Test-Enhanced Learning”
	Multiple Fields	(TrueLearn, n.d.)	“Goodbye, Q-Banks. Hello, SmartBanks.”

## Data Availability

Not applicable.

## Abbreviations

The following abbreviations are used in this manuscript:

LASSI	Learning and Study Strategies Inventory
LCME	Liaison Committee on Medical Education
MCAT	Medical College Admission Test
MELS	Medical Education Learning Specialist(s)
NBME	National Board of Medical Examiners
PBL	Problem-Based Learning
USMLE	United States Medical Licensing Examination
